# Transactions of the Medical Society of the State of New York

**Published:** 1840-11

**Authors:** 


Art. VII.—*Transactions of the Medical Society of the State of New York.*—Parts II and III, Vol. IV.

The Medical Society of the State of New York is a legal organization of the medical profession, designed to protect the community against the impositions of unqualified practitioners, and to provide for the suitable education of those who aspire to the honorable but responsible office of healing the maladies of their fellow-citizens. And surely no object can be presented to the mind of an enlightened and patriotic legislator, which should more powerfully enchain his attention, or more vigorously call forth all his energies for its attainment. Such an one must feel, after midnight vigils spent in devising remedies for the over-reaching of the avaricious and crafty, where dollars and cents alone are involved, that his solicitude would have been more worthily bestowed in guarding against the wiles of quackery, which filches not only the purse, but the health and lives of its dupes.

The State Medical Society of New York, which owes its existence to wise and provident legislation, is composed of delegates from county medical associations, that have each a corporate capacity, and are empowered by law to grant licenses to practice to such as have complied with specified conditions, and are found qualified after an examination by a board of censors. The County and State Societies hold regular meetings for the mutual improvement of their members, and to contribute their quota to the advancement of science. The State Society convenes annually at the capitol, in the month of February, and publishes, in the shape of *Transactions*, the avails of the systematic and stimulated labors of all within the limits of its jurisdiction.

Parts No. II and III of vol. IV, which are now before us, contain Addresses delivered on different occasions, and Essays on various subjects, together with abstracts of the Proceedings of the Society at its annual sessions in 1839, 1840. The following enumeration will convey an idea of their genral character:—1. *Annual Address on Quackery*, delivered before the Medical Society of the State of New York, February 6th, 1839, by Laurens Hull, M. D., President of the Society. 2. *Address on Spinal Disease*, delivered before the Tompkins County Medical Society in 1833, by Dr. A. Church, President of the Society. 3. *Address on Quackery*, delivered before the Tompkins County Medical Society in 1834, by Dr. A. Church, President of the Society. 4. *Address on Medical Societies*, delivered before the Tompkins County Medical Society in 1835, by the late Daniel D. Page, M. D., President of the Society. 5. *Address on Observation and Attention*, delivered before the Tompkins County Medical Society in 1836, by the late Daniel D. Page, M. D., President of the Society. 6. *On Suicide and its Increase in the present day.* By John Ludw Casper, M. D., Professor in the University of Berlin, etc. Translated from the German by Horace. B. Webster. 7. *Statistics of the Medical Colleges of the United States.* By T. Romeyn Beck, M. D. 8. *Annual Address on Improvement in Medicine*, delivered before the Medical Society of the State of New York, February 5th, 1840. by Laurens Hull, M. D., President of the Society. 9. *Report of a Committee of the State Medical Society on the Subject of Medical Education.* Presented February 4th, 1840. 10. *On Diseases of the Spinal Column, their Causes, Diagnosis, History, and best mode of Treatment; being the* Prize Dissertation *for* 1840, by Nathan S. Davis, M. D., of Binghampton, Broome county, New York. "L'ami de nature." 11. *Address delivered before the* Chenango *County Medical Society*, October 8th, 1838, by Dr. Wm. D. Purple, President of the Society. 12. *Case of Sudden Death from Rupture of the Spermatic Vein.* By James M'Naughton, M. D., of Albany.

From this enumeration it will be seen, that the bill of fare offered us is sufficiently multifarious: we shall taste but few of its dishes at present, and are admonished by our limits to make a very temperate repast from even these. The *Report on Medical Education* by Drs. Beck, Manly and M'Call, is a very able and interesting document. It opens with a retrospect of the statutes which have been enacted, at different times, by the Legislature of New York, for the purpose of regulating the practice of Physic and Surgery in that Commonwealth. From the historical sketch the authors have drawn we gather that the evils of quackery claimed the attention of the Legislature at a very early period; that a series of acts were successively passed to insure competent qualification in those who should offer their services as practitioners of medicine; that the standard of qualification was gradually raised until the present system was established. According to its provisions, a student is required to study medicine four years with some physician and surgeon duly authorized by law to practice his profession, and at the end of his pupilage submit to an examination by a board of censors, before he can be allowed to practice, and no person is to be licensed until he has arrived at the age of twenty-one years. The law, however, admits the following exceptions; one year might be deducted from the term of study, provided the pupil had attended to *classical studies* for that period after the age of sixteen; and again, "*attendance on a complete course of lectures delivered by each of the professors, on all the branches of medical science in either* of *the medical colleges or institutions of this State or elsewhere*," might be excepted in lieu of one year's study.

The design of the first of these exceptions, it is evident, is to hold out an inducement to students to acquire a proper preliminary education. The committee address themselves to the inquiry whether this is all that should be done to encourage preparatory education, and after comparing the medical with the legal and clerical profession, they arrive at the conclusion, that something more is necessary, and propose the requisition that the study of medicine be determined upon at the age, say of sixteen, and that two years shall be devoted to classical studies, *or the higher branches of English studies equivalent to them.* Any feasible method of securing a proper amount of preliminary education to those who are destined for the medical profession, shall ever receive our hearty concurrence. The illiteracy of too many of those, who are thrusting themselves into our ranks, is a reproach and degradation to our profession, and has given currency to the belief that a dolt, who is too stupid for the bar or the pulpit, will do admirably well for medicine. But it does not occur to us that the object can be best attained by legal enactments; we should rather appeal to the *vis conservatrix* that is inherent in the profession itself, but has been shamefully permitted to slumber until our ranks exhibit a motley assemblage of ignorance and knowledge, talents and stupidity. How happens it that ministers of several denominations in this country are, as a class, better educated than physicians? Simply because the church has taken hold of the subject in the right spirit, and, under the conviction that a thorough course of training will augment the usefulness and respectability, nay, is indispensable to the success of the ministry, has prescribed a course of studies, to which all candidates are obliged to conform before they can be licensed to preach. In like manner, let our medical colleges exact a prescribed amount of attainments in classical learning and the higher branches of English studies, as a pre-requisite to graduation, and the work of purification will have been commenced, which, if faithfully persevered in, will eventuate in the restoration of the medical to its proper rank among the learned professions. We see no other remedy for the evil complained of. It is repugnant to the genius of our government, as it is to the temper and spirit of the governed, that the law should take cognizance of what may as well be entrusted to the voluntary action of those who are chiefly interested. It is the interest of society to be protected against charlatanry, and it is therefore incumbent on the legislature to pass laws for its extermination; but those who are to pronounce on the qualification of candidates for license or graduation can best prescribe and enforce the course of studies, that shall be complied with, and they alone are the judges of the actual proficiency the pupil has made.

We have extended our remarks on this topic farther than we intended, and must turn to notice, very cursorily, two of the papers in the collection before us, which appear to possess the greatest practical interest; these are Dr. Church's *Address on Spinal Disease*, and Dr. Davis's *Essay on Diseases of the Spinal Column.*

Considering the extent and connexions of the spinal cord—running from one extremity of the trunk to the other, connected with the cerebral nerves and ganglionic system, and supplying nerves to the upper and lower extremities, indeed to almost the whole muscular system, Dr. Church is persuaded that it exercises a considerable influence over a great variety of diseases, and that it has been too much overlooked by physicians. While he acknowledges his indebtedness to the valuable little work of *Thomas Pridgen Teale*, for the first hints which he received respecting spinal diseases, Dr. Church claims to have successively treated a case on the principles laid down by that writer, just previous to the appearance of his treatise. The discovery of the nature of the case, he modestly avers, was rather the result of accident than of any sagacity of his. The case alluded to was that of a lady aged about 24 or 26 years, who was reported to have suffered from severe pulmonary inflammation for several months previously, for which she had been bled repeatedly, and used other debilitating remedies.

"When I first saw her she scarcely complained of anything except debility, and occasionally a peculiar indescribable lassitude, which would suddenly come over her, when she was unable to support herself and was obliged to take to her bed where she was confined most of her time. On examination, the different functions appeared to be performed with the regularity of perfect health, pulse perfectly natural, tongue clear, appetite good, bowels and catamenia regular and skin natural. She complained of a tightness about the chest and a difficulty of breathing which occasionally came on in the evening and lasted for a short time and disappeared. I was inclined to attribute these symptoms to debility and accordingly prescribed tonics. After a trial of about ten days they had no effect whatever, and I was at a loss what to do. From some cause my attention was directed to the spine, on examining which, I found three of the dorsal vertebræ quite tender to the touch, and pressure upon them caused a stricture and tightness of the chest. I suspected this was in some way connected with the disease, if not a principal cause of it, and directed a blister to be applied to the part. I had the satisfaction to see the most decided relief result from this, and following it with the use of quinine and Dover's powders, together with tartar emetic ointment to the local affection for some time, the patient was restored to a tolerable state of health. I ought to have observed that during her convalescence, her feet and ankles swelled and became painful, attended with pain in the loins; this however, subsided without any particular treatment. I have conjectured that the case of this lady might have been originally an acute neuralgia of the chest, and not an inflammation. I have met with such cases in my practice and have generally found them very much relieved by bleeding, for a few hours, but the pain and stricture almost invariably return very soon, and if the bleeding is persisted in, or repeated often, the pain becomes aggravated at every repetition, until the patient is brought to the borders of the grave, and I have reason to believe that such treatment has proved fatal in more than one instance. When a delicate female, or any one in feeble health, is suddenly attacked with very acute pain of the side, if this pain is quickly relieved by bleeding, and returns again in a few hours after each bleeding, we ought to be extremely cautious lest we be imposed upon by the semblance of genuine inflammation, when nothing but neuralgia is the cause of it, which is certainly aggravated by such depletion."

Dr. Church has met with a number of cases of disordered digestion, attended with soreness of the upper cervical vertebræ, which, he thought, had a very considerable influence over the disease, and sometimes seemed to be the principal cause of it. He relates the following very striking case:—

"A gentleman about forty-five years of age consulted me about two years ago, on account of a disordered state of the stomach, attended with a great deal of palpitation of the heart. He was greatly troubled with water-brash, tongue furred, bowels irregular, occasionally some fever. He had been troubled with the most of these sysmptoms for seven years, more particularly the palpitation and water-brash. When I first saw him the palpitation was alarming, and I had little doubt that he had an organic affection of the heart. I examined the cervical vertebræ, and found them very tender, particularly the upper ones. I concluded to adopt the plan of blistering the spine, together with alterative doses of blue pill, and a tonic of pulv. columbo. On seeing the patient two or three days after, I had the satisfaction of learning that the palpitation and water-brash had nearly disappeared immediately after the blister had drawn. His health rapidly improved from this time without interruption, exept an attack of ague and fever a short time afterwards, which was cured by quinine."

There is, he thinks, good reason to believe that sick-headache, which so often baffles every effort to cure or even to relieve it, is often an affection of the same part of the nervous system; he does not recollect an instance of habitual sick-headache, that has fallen under his observation, in which there was not soreness in the upper cervical vertebræ, and he has treated the complaint with blisters and irritants to the nape of the neck, obtaining thereby not only temporary relief but a radical cure.

We can add our testimony to the relief derived from irritants, particularly sinapisms over the cervical vertebræ, in this distressing affection, although we have not pursued this method of treatment with a view to a raidical cure, and cannot therefore pronounce upon its efficacy from observation. Three cases are related by Dr. Church, in illustration of the treatment recommended ; the following is one of them :

" A married lady of twenty-six years of age, had been for several years severely afflicted with what she called the blind headache. It came on once in a few months, commencing with sickness of the stomach, headache and giddiness to such a degree, that she was obliged to remain in bed constantly, and every attempt to raise herself in bed induced blindness for the moment. The remedies formerly used had done but little to alleviate the complaint, and she had come to the conclusion there was no remedy for it. I accidentally saw her while confined with one of these attacks, and inquired into her symptoms, particularly whether there was any soreness in the neck. I found there was a great degree, and on particular inquiry learned of her that she was habitually subject to a slight stiffness of the neck, not however to such a degree as to excite much attention. Under these circumstances I strenuously recommended a blister to the nape of the neck, and without any other remedy the disorder abated immediately, and she was well of her complaint by the time the blister had healed. I deemed it advisable to use stimulating liniment to the neck for some time as a precaution against a return of it. This occurred two years and a half ago, and the complaint has never returned."

Dr. Church closes his Address with the rehearsal of three cases of rheumatism in which spinal irritation or inflammation existed, and in which cups, blisters and stimulating liniments appeared to afford considerable relief; but as these are not particularly striking and less satisfactory than some others that have been published, especially those of Dr. John K. Mitchell in the American Journal of Medical Sicences for August, 1833, we forbear to quote from them.

Dr. Davis' essay on diseases of the spinal column is a much more elaborate production than that of Dr. Church; its scope, also, is more extensive, embracing not only irritation but inflammation of the spinal cord, and curvatures of the spinal column. It is in fact a monograph of the whole subject of spinal diseases, evidently prepared with care, and is a good digest of what has been written by the most esteemed authors, interspersed with some very interesting observations of his own. In the closing chapter of his essay, *on spinal irritation*, the only one which our limits will permit us to notice, the author very properly cautions against ascribing too wide a range to the morbid influences of this peculiar condition of the medulla spinalis,—which, according to some writers, is the first link, the grand starting point of every disease. To enforce this caution, he relates the following instructive case:

"Not long since I was called to visit a young lady who had been scarcely able to leave her room except in a carriage for four or five years. She was considerably emaciated, with a languid countenance, tongue rather *red* but moist, pulse languid and feeble, bowels generally slightiy costive, sometimes relaxed, with frequent severe pain after indulging in too much food, appetite moderate, much pain along the course of the spine, and especially in the lumbar region, tenderness to pressure over the spinous processes of the inferior dorsal and upper lumbar vertebræ, great excitability, as well as debility of the whole system, with frequent palpitations of the heart, and a feeling of great languor and indisposition to exertion.

"This lady had been attended at different times, by a number of medical gentlemen, some of them of high standing in their profession. Her complaint had been considered one of general debility, *nervous irritation*, dyspepsia, or consumption.

"She had used accordingly and in succession tonics, chalybeates, dieting, very slight *frictions* with *tartar emetic ointment* over the *spine*, mercury to salivation, &c. She had once or twice resorted to the most celebrated watering places in our country, but all with no permanent benefit, and her health on the whole continued to fail, Not being satisfied in regard to the nature of the pain in the back, I determined on making a more thorough examination. This was attended with some difficulty owing to the extreme delicacy of the patient. At length however, an old and experienced accoucheur, was permitted to make the desired examination.—And to *his* surprise "the os uteri was found low down in the vagina, and the fundus of the uterus thrown back toward the sacrum;" constituting a considerable degree of *prolapsus uteri* with partial *retroversion.* No cause for this displacement could be ascertained except the pernicious habit of *tight lacing*, and perhaps a little too free exercise in ascending a flight of stairs to take care of a sick relative, a short time previous to the commencement of her illness. And yet to any one acquainted with the extensive influence which this displacement exerts over the other organs of the system, it will afford an ample explanation of the preceding symptoms. And I scarcely need to add that the application of a proper mode of treatment, designed principally for remedying the uterine affection, has already afforded much relief."

Nevertheless Dr. Davis is well convinced that morbid irritability or irritation of the spinal cord may give rise to a great variety of distressing functional disorders in other organs, which will yield to no other treatment than counter-irritation over the spine. H. M.

